# Crystal structures of methyl 3-phenyl-4,5-di­hydro-1*H*,3*H*-benzo[4,5]imidazo[2,1-*c*][1,4]oxazepine-4-carboxyl­ate and methyl 1-methyl-3-phenyl-4,5-di­hydro-1*H*,3*H*-benzo[4,5]imidazo[2,1-*c*][1,4]oxazepine-4-carboxyl­ate

**DOI:** 10.1107/S1600536814021655

**Published:** 2014-10-08

**Authors:** J. Govindaraj, R. Raja, M. Suresh, R. Raghunathan, A. SubbiahPandi

**Affiliations:** a Department of Physics, Pachaiyappa’s College for Men, Kanchipuram 631 501, India; b Department of Physics, Presidency College (Autonomous), Chennai 600 005, India; c Department of Organic Chemistry, University of Madras, Guindy campus, Chennai 602 025, India

**Keywords:** crystal structure, oxazepine, benzimidazole, angiogenesis, natural products.

## Abstract

The title compounds, C_19_H_18_N_2_O_3_, (I), and C_20_H_20_N_2_O_3_, (II), differ only by a methyl substituent on the seven-membered oxazepine ring in (II). In both compounds, these rings have a twist-chair conformation. The phenyl ring makes a dihedral angle of 73.42 (10)° with the benzimidazole ring system mean plane (r.m.s. deviation = 0.015 Å) in (I) and 83.07 (7)° in (II) (r.m.s. deviation = 0.026 Å). The methyl carboxyl­ate groups are planar to within 0.031 (2) in (I) and 0.003 (2) Å in (II). They are inclined to the phenyl and benzimidazole ring system by 33.78 (16) and 87.56 (14)°, respectively, in (I) and by 53.04 (12) and 60.22 (11)°, respectively, in (II). In the crystal of (I), mol­ecules stack in a herringbone fashion and are linked by C—H⋯O hydrogen bonds, forming chains along [100]. In the crystal of (II), there are no significant inter­molecular inter­actions present.

## Abstract 2

In two benzo[4,5]imidazo[2,1-*c*][1,4]oxazepine-4-carboxyl­ates, the seven-membered oxazepane rings both have a twist-chair conformation. The dihedral angle between the phenyl ring and the benzimidazole ring system is significantly smaller in one of the compounds, *viz.* 73.42 (10) compared to 83.07 (17)°.

## Chemical context   

Fused oxazepinone derivatives have attracted considerable attention owing to their promising biological activities, such as anti­cancer, anti-HIV, anti­depressant and anti­tumor activities (Liu *et al.*, 2011 ▶). Tumor growth requires the support of an associated blood supply, making tumor vasculature a potential target for anti­cancer therapy. This principle has inspired decades of research into the pathways of angiogenesis (the formation of new blood vessels), leading to the identification of a family of vascular endothelial growth factors (VEGFs) that stimulate this process (Edwards *et al.*, 2011 ▶). Seven-membered oxygen heterocycles are ubiquitous in natural products and show a wide spectrum of biological activity (Bera *et al.*, 2014 ▶).
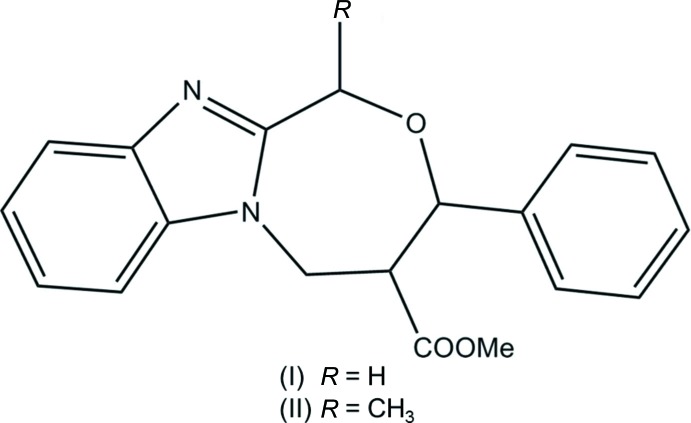


## Structural commentary   

**Figure 1:**
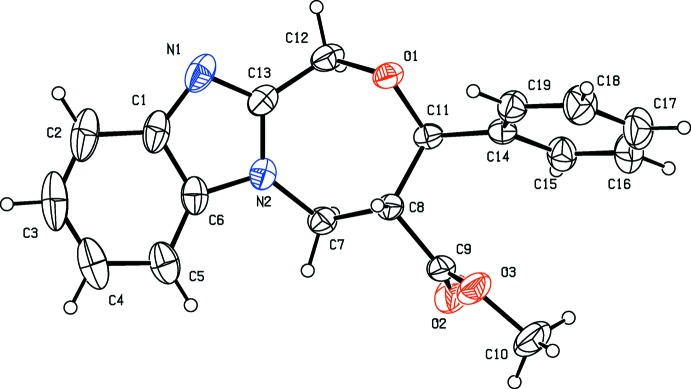
The mol­ecular structure of compound (I), with atom labelling. Displacement ellipsoids are drawn at the 30% probability level.

The mol­ecular structure of compound (I) is illustrated in Fig. 1 ▶. The C1—N1—C13 bond angle is 105.2 (2)°. The seven-membered oxazepine ring (O1/N2/C7/C8/C11–C13) has a twist-chair conformation, as can be evidenced by the torsion angles C12—C13—N2—C7 = −3.2 (3) and C8—C11—O1—C12 = −78.33 (18)°. The phenyl ring (C14–C19) is inclined to the benzimidazole ring system [N1/N2/C1–C6/C13; r.m.s. deviation = 0.026 Å] by 73.42 (10)°. The methyl carboxyl­ate group (C9/O2/O3/C10) is planar to within 0.031 (2) Å and is inclined to the phenyl ring and the benzimidazole ring system by 33.78 (16) and 87.56 (14)°, respectively.

**Figure 2:**
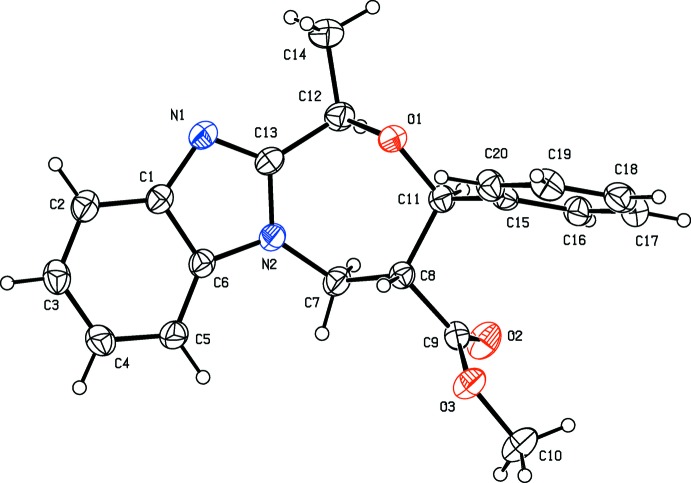
The mol­ecular structure of compound (II), with atom labelling. Displacement ellipsoids are drawn at the 30% probability level.

The mol­ecular structure of compound (II) is illustrated in Fig. 2 ▶. The C1—N1—C13 bond angle of 104.27 (15)°. The seven-membered oxazepine ring (O1/N2/C7/C8/C11–C13) also has a twist-chair conformation, with torsion angles C12—C13—N2—C7 = −6.6 (3) and C8—C11—O1—C12 = −74.17 (18)°.

The principle difference in the two compounds concerns the orientation of the phenyl ring (C15–C20) with respect to the benzimidazole ring system [N1/N2/C1–C6/C13; r.m.s. deviation = 0.026 Å]. In (II), this angle is 83.07 (17)° considerably larger than the same angle in (I), *viz* 73.42 (10)°. Here the methyl carboxyl­ate group (C9/O2/O3/C10), planar to within 0.003 (2) Å, is inclined to the phenyl ring and the benzimid­azole ring system by 53.04 (12) and 60.22 (11)°, respectively. These angles are also very different to those observed in compound (I), *viz* 33.78 (16) and 87.56 (14)°, respectively.

## Supra­molecular features   

**Figure 3:**
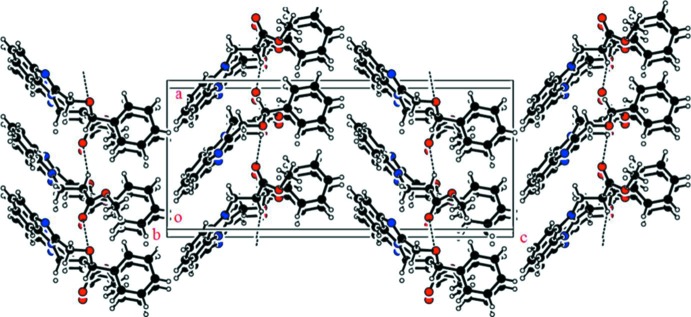
A view along the *b* axis of the crystal packing of compound (I). The hydrogen bonds are shown as dashed lines (see Table 1 ▶ for details).

In the crystal of (I), mol­ecules stack in a herringbone fashion and are linked by C—H⋯O hydrogen bonds, forming chains along the *a-*axis direction (Table 1 ▶ and Fig. 3 ▶).

In the crystal of (II), there are no significant inter­molecular inter­actions present.

## Database survey   

In the Cambridge Structural Database (Version 5.35, last update May 2014; Groom & Allen, 2014 ▶) there are a large number of compounds containing an oxazepine-type ring, but only one entry was found for such a ring fused to a benz­imid­azole unit. This compound, 1*H*,3*H*-[1,4][4,3-a]benzimid­azole (UQILOW; Zhang *et al.*, 2011 ▶), has an oxazepino ring with a C=C bond in the seven-membered ring.

## Synthesis and crystallization   

A mixture of *Z*-methyl-2-(bromo­meth­yl)-3-phenyl­acrylate (1.0 mol) and (1*H*-benzo[*d*]imidazole-2-yl)methanol (1.1 mol) for (I), but (1*H*-benzo[*d*]imidazole-2-yl)ethanol (1.1 mol) for (II), together with CS_2_CO_3_ (1 mol) in CH_3_CN (10 ml) was stirred for 8 h. After completion of the reactions, monitored by TLC, the solvents were evaporated under reduced pressure. The residues were diluted with ethyl acetate then washed with brine and water. The organic layers were separated and the residues were subjected to column chromatography using ethyl acetate and hexane (2:8) as eluent. The products were dissolved in chloro­form and heated for 2 min. The resulting solutions were subjected to crystallization by slow evaporation of the solvent for 48 h resulting in the formation of colourless block-like crystals of compounds (I) and (II).

## Refinement   

Crystal data, data collection and structure refinement details are summarized in Table 2 ▶.

 In both compunds, the C-bound H atoms were positioned geometrically and allowed to ride on their parent atoms: C—H = 0.93–0.98 Å with *U*_iso_(H) = .2*U*_eq_(C) or 1.5*U*_eq_(Cmethyl).

## Supplementary Material

Crystal structure: contains datablock(s) global, I, II. DOI: 10.1107/S1600536814021655/su2789sup1.cif

Structure factors: contains datablock(s) I. DOI: 10.1107/S1600536814021655/su2789Isup2.hkl

Structure factors: contains datablock(s) II. DOI: 10.1107/S1600536814021655/su2789IIsup3.hkl

Supporting information file. DOI: 10.1107/S1600536814021655/su2789Isup4.cml

Supporting information file. DOI: 10.1107/S1600536814021655/su2789IIsup5.cml

CCDC references: 1027182, 1027183

Additional supporting information:  crystallographic information; 3D view; checkCIF report

## Figures and Tables

**Table 1:** Hydrogen-bond geometry (, ) for (I)

*D*H*A*	*D*H	H*A*	*D* *A*	*D*H*A*
C8H8O2^i^	0.98	2.35	3.225(2)	148

Symmetry code: (i) 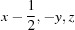.

**Table 2:** Experimental details

	(I)	(II)
Crystal data		
Chemical formula	C_19_H_18_N_2_O_3_	C_20_H_20_N_2_O_3_
*M*_r_	322.35	336.38
Crystal system, space group	Orthorhombic, *P* *c* *a*2_1_	Orthorhombic, *P*2_1_2_1_2_1_
Temperature (K)	293	293
*a*, *b*, *c* ()	9.9238(13), 7.3322(10), 23.028(3)	9.1115(7), 9.6470(8), 19.4856(15)
*V* (^3^)	1675.6(4)	1712.8(2)
*Z*	4	4
Radiation type	Mo *K*	Mo *K*
(mm^1^)	0.09	0.09
Crystal size (mm)	0.21 0.19 0.18	0.21 0.19 0.18

Data collection		
Diffractometer	Bruker *SMART* APEXII CCD	Bruker *SMART* APEXII CCD
Absorption correction	Multi-scan (*SADABS*; Bruker, 2008 ▶)	Multi-scan (*SADABS*; Bruker, 2008 ▶)
*T*_min_, *T*_max_	0.982, 0.984	0.981, 0.984
No. of measured, independent and observed [*I* > 2(*I*)] reflections	15223, 3639, 2782	30423, 3847, 2958
*R*_int_	0.023	0.043
(sin /)_max_ (^1^)	0.637	0.645

Refinement		
*R*[*F* ^2^ > 2(*F* ^2^)], *wR*(*F* ^2^), *S*	0.036, 0.089, 1.04	0.037, 0.089, 1.06
No. of reflections	3587	3844
No. of parameters	217	226
No. of restraints	1	0
H-atom treatment	H-atom parameters constrained	H-atom parameters constrained
_max_, _min_ (e ^3^)	0.13, 0.13	0.15, 0.13

Computer programs: *APEX2* and *SAINT* (Bruker, 2008 ▶), *SHELXS97* and *SHELXL97* (Sheldrick, 2008 ▶) and *PLATON* (Spek, 2009 ▶).

## supplementary crystallographic information

### Crystal data

**Table:**

C_20_H_20_N_2_O_3_	*F*(000) = 712
*M**_r_* = 336.38	*D*_x_ = 1.304 Mg m^−^^3^
Orthorhombic, *P*2_1_2_1_2_1_	Mo *K*α radiation, λ = 0.71073 Å
Hall symbol: P 2ac 2ab	Cell parameters from 2958 reflections
*a* = 9.1115 (7) Å	θ = 2.1–27.3°
*b* = 9.6470 (8) Å	µ = 0.09 mm^−^^1^
*c* = 19.4856 (15) Å	*T* = 293 K
*V* = 1712.8 (2) Å^3^	Block, colourless
*Z* = 4	0.21 × 0.19 × 0.18 mm

### Data collection

**Table:**

Bruker SMART APEXII CCD diffractometer	3847 independent reflections
Radiation source: fine-focus sealed tube	2958 reflections with *I* > 2σ(*I*)
Graphite monochromator	*R*_int_ = 0.043
ω and φ scans	θ_max_ = 27.3°, θ_min_ = 2.1°
Absorption correction: multi-scan (*SADABS*; Bruker, 2008)	*h* = −11→11
*T*_min_ = 0.981, *T*_max_ = 0.984	*k* = −12→12
30423 measured reflections	*l* = −25→25

### Refinement

**Table:**

Refinement on *F*^2^	Primary atom site location: structure-invariant direct methods
Least-squares matrix: full	Secondary atom site location: difference Fourier map
*R*[*F*^2^ > 2σ(*F*^2^)] = 0.037	Hydrogen site location: inferred from neighbouring sites
*wR*(*F*^2^) = 0.089	H-atom parameters constrained
*S* = 1.06	*w* = 1/[σ^2^(*F*_o_^2^) + (0.0335*P*)^2^ + 0.3064*P*] where *P* = (*F*_o_^2^ + 2*F*_c_^2^)/3
3844 reflections	(Δ/σ)_max_ < 0.001
226 parameters	Δρ_max_ = 0.15 e Å^−^^3^
0 restraints	Δρ_min_ = −0.13 e Å^−^^3^

### Special details

**Table:**

Geometry. All e.s.d.'s (except the e.s.d. in the dihedral angle between two l.s. planes) are estimated using the full covariance matrix. The cell e.s.d.'s are taken into account individually in the estimation of e.s.d.'s in distances, angles and torsion angles; correlations between e.s.d.'s in cell parameters are only used when they are defined by crystal symmetry. An approximate (isotropic) treatment of cell e.s.d.'s is used for estimating e.s.d.'s involving l.s. planes.
Refinement. Refinement of *F*^2^ against ALL reflections. The weighted *R*-factor *wR* and goodness of fit *S* are based on *F*^2^, conventional *R*-factors *R* are based on *F*, with *F* set to zero for negative *F*^2^. The threshold expression of *F*^2^ > σ(*F*^2^) is used only for calculating *R*-factors(gt) *etc*. and is not relevant to the choice of reflections for refinement. *R*-factors based on *F*^2^ are statistically about twice as large as those based on *F*, and *R*- factors based on ALL data will be even larger.

### Fractional atomic coordinates and isotropic or equivalent isotropic displacement parameters (Å^2^)

**Table:**

	*x*	*y*	*z*	*U*_iso_*/*U*_eq_	
C1	0.43553 (19)	−0.09161 (19)	0.06180 (9)	0.0410 (4)	
C2	0.4623 (2)	−0.1711 (2)	0.11986 (9)	0.0490 (5)	
H2	0.5290	−0.1422	0.1530	0.059*	
C3	0.3875 (2)	−0.2934 (2)	0.12673 (10)	0.0525 (5)	
H3	0.4044	−0.3483	0.1652	0.063*	
C4	0.2871 (2)	−0.3375 (2)	0.07775 (10)	0.0541 (5)	
H4	0.2390	−0.4216	0.0840	0.065*	
C5	0.2572 (2)	−0.2600 (2)	0.02042 (10)	0.0501 (5)	
H5	0.1893	−0.2889	−0.0122	0.060*	
C6	0.33290 (19)	−0.13700 (19)	0.01370 (9)	0.0411 (4)	
C7	0.2428 (2)	−0.0325 (2)	−0.09788 (9)	0.0471 (4)	
H7A	0.1888	0.0541	−0.0983	0.057*	
H7B	0.1719	−0.1074	−0.0958	0.057*	
C8	0.32954 (19)	−0.04495 (18)	−0.16429 (8)	0.0368 (4)	
H8	0.3938	−0.1262	−0.1614	0.044*	
C9	0.21833 (18)	−0.06790 (19)	−0.22047 (9)	0.0393 (4)	
C10	0.1375 (3)	−0.2192 (3)	−0.30710 (11)	0.0694 (7)	
H10A	0.1643	−0.3063	−0.3274	0.104*	
H10B	0.1394	−0.1481	−0.3415	0.104*	
H10C	0.0404	−0.2259	−0.2882	0.104*	
C11	0.42285 (19)	0.08308 (18)	−0.18173 (9)	0.0394 (4)	
H11	0.3580	0.1634	−0.1871	0.047*	
C12	0.4747 (2)	0.17200 (19)	−0.06734 (9)	0.0487 (5)	
H12	0.3866	0.2268	−0.0771	0.058*	
C13	0.4367 (2)	0.05805 (18)	−0.01878 (9)	0.0429 (4)	
C14	0.5926 (3)	0.2654 (2)	−0.03933 (12)	0.0712 (7)	
H14A	0.6138	0.3370	−0.0721	0.107*	
H14B	0.6797	0.2122	−0.0308	0.107*	
H14C	0.5594	0.3066	0.0027	0.107*	
C15	0.50539 (18)	0.06069 (16)	−0.24765 (10)	0.0379 (4)	
C16	0.4532 (2)	0.11214 (19)	−0.30893 (9)	0.0465 (4)	
H16	0.3662	0.1626	−0.3098	0.056*	
C17	0.5294 (2)	0.0891 (2)	−0.36909 (10)	0.0552 (5)	
H17	0.4928	0.1237	−0.4102	0.066*	
C18	0.6573 (2)	0.0165 (2)	−0.36889 (11)	0.0566 (5)	
H18	0.7087	0.0025	−0.4095	0.068*	
C19	0.7098 (2)	−0.0359 (2)	−0.30812 (11)	0.0552 (5)	
H19	0.7969	−0.0861	−0.3076	0.066*	
C20	0.63416 (19)	−0.01449 (19)	−0.24811 (11)	0.0488 (4)	
H20	0.6702	−0.0511	−0.2073	0.059*	
N1	0.50049 (16)	0.03094 (16)	0.04017 (7)	0.0458 (4)	
N2	0.33366 (16)	−0.03703 (16)	−0.03703 (7)	0.0447 (4)	
O1	0.52857 (14)	0.11254 (12)	−0.12973 (6)	0.0446 (3)	
O2	0.11936 (15)	0.01004 (15)	−0.23210 (7)	0.0603 (4)	
O3	0.23993 (15)	−0.18523 (13)	−0.25338 (7)	0.0535 (4)	

### Atomic displacement parameters (Å^2^)

**Table:**

	*U*^11^	*U*^22^	*U*^33^	*U*^12^	*U*^13^	*U*^23^
C1	0.0375 (9)	0.0489 (10)	0.0367 (9)	0.0039 (8)	0.0027 (8)	−0.0057 (8)
C2	0.0386 (9)	0.0684 (13)	0.0399 (10)	0.0008 (10)	0.0022 (8)	−0.0003 (9)
C3	0.0456 (11)	0.0683 (14)	0.0435 (11)	0.0030 (10)	0.0077 (9)	0.0088 (10)
C4	0.0544 (12)	0.0584 (12)	0.0494 (12)	−0.0093 (10)	0.0118 (10)	0.0019 (10)
C5	0.0489 (11)	0.0634 (12)	0.0381 (10)	−0.0133 (10)	0.0035 (9)	−0.0053 (10)
C6	0.0379 (9)	0.0524 (10)	0.0330 (9)	−0.0016 (8)	0.0053 (8)	−0.0036 (8)
C7	0.0405 (9)	0.0627 (12)	0.0381 (10)	−0.0040 (9)	−0.0042 (8)	−0.0007 (9)
C8	0.0354 (9)	0.0372 (9)	0.0379 (9)	0.0036 (7)	−0.0033 (7)	0.0022 (8)
C9	0.0378 (9)	0.0434 (10)	0.0368 (9)	0.0017 (8)	0.0014 (7)	0.0052 (8)
C10	0.0745 (15)	0.0751 (15)	0.0585 (13)	0.0034 (13)	−0.0242 (12)	−0.0165 (12)
C11	0.0416 (10)	0.0356 (9)	0.0409 (10)	0.0032 (8)	−0.0054 (8)	0.0024 (8)
C12	0.0612 (12)	0.0375 (9)	0.0475 (11)	0.0008 (9)	−0.0051 (9)	−0.0054 (8)
C13	0.0448 (10)	0.0434 (10)	0.0404 (10)	0.0018 (8)	−0.0019 (8)	−0.0085 (8)
C14	0.0943 (18)	0.0528 (13)	0.0664 (14)	−0.0206 (12)	−0.0132 (13)	−0.0048 (11)
C15	0.0378 (8)	0.0311 (8)	0.0447 (9)	−0.0030 (7)	−0.0022 (8)	0.0036 (8)
C16	0.0441 (10)	0.0442 (10)	0.0511 (11)	0.0013 (9)	−0.0054 (9)	0.0079 (9)
C17	0.0622 (13)	0.0578 (12)	0.0456 (11)	−0.0081 (11)	−0.0035 (10)	0.0086 (10)
C18	0.0603 (13)	0.0543 (12)	0.0551 (13)	−0.0113 (11)	0.0128 (10)	−0.0035 (10)
C19	0.0465 (11)	0.0511 (12)	0.0681 (14)	0.0030 (9)	0.0072 (10)	0.0021 (10)
C20	0.0457 (10)	0.0489 (10)	0.0518 (11)	0.0047 (9)	−0.0016 (9)	0.0105 (10)
N1	0.0487 (9)	0.0488 (9)	0.0399 (8)	−0.0015 (8)	−0.0035 (7)	−0.0068 (7)
N2	0.0428 (8)	0.0547 (9)	0.0365 (8)	−0.0070 (7)	−0.0034 (7)	0.0004 (7)
O1	0.0466 (7)	0.0437 (7)	0.0435 (7)	−0.0041 (6)	−0.0056 (6)	−0.0017 (6)
O2	0.0539 (8)	0.0677 (9)	0.0592 (9)	0.0230 (8)	−0.0151 (7)	−0.0061 (7)
O3	0.0579 (8)	0.0492 (7)	0.0535 (8)	0.0079 (7)	−0.0176 (7)	−0.0091 (7)

### Geometric parameters (Å, º)

**Table:**

C1—N1	1.388 (2)	C10—H10C	0.9600
C1—C2	1.388 (3)	C11—O1	1.427 (2)
C1—C6	1.394 (2)	C11—C15	1.504 (2)
C2—C3	1.369 (3)	C11—H11	0.9800
C2—H2	0.9300	C12—O1	1.431 (2)
C3—C4	1.389 (3)	C12—C13	1.491 (3)
C3—H3	0.9300	C12—C14	1.504 (3)
C4—C5	1.371 (3)	C12—H12	0.9800
C4—H4	0.9300	C13—N1	1.313 (2)
C5—C6	1.379 (3)	C13—N2	1.360 (2)
C5—H5	0.9300	C14—H14A	0.9600
C6—N2	1.381 (2)	C14—H14B	0.9600
C7—N2	1.447 (2)	C14—H14C	0.9600
C7—C8	1.521 (2)	C15—C16	1.378 (3)
C7—H7A	0.9700	C15—C20	1.379 (2)
C7—H7B	0.9700	C16—C17	1.380 (3)
C8—C9	1.508 (2)	C16—H16	0.9300
C8—C11	1.537 (2)	C17—C18	1.360 (3)
C8—H8	0.9800	C17—H17	0.9300
C9—O2	1.196 (2)	C18—C19	1.374 (3)
C9—O3	1.316 (2)	C18—H18	0.9300
C10—O3	1.440 (2)	C19—C20	1.373 (3)
C10—H10A	0.9600	C19—H19	0.9300
C10—H10B	0.9600	C20—H20	0.9300
			
N1—C1—C2	130.01 (17)	O1—C11—H11	109.0
N1—C1—C6	110.46 (15)	C15—C11—H11	109.0
C2—C1—C6	119.49 (17)	C8—C11—H11	109.0
C3—C2—C1	117.90 (18)	O1—C12—C13	108.86 (14)
C3—C2—H2	121.0	O1—C12—C14	107.67 (17)
C1—C2—H2	121.0	C13—C12—C14	112.14 (17)
C2—C3—C4	121.64 (19)	O1—C12—H12	109.4
C2—C3—H3	119.2	C13—C12—H12	109.4
C4—C3—H3	119.2	C14—C12—H12	109.4
C5—C4—C3	121.59 (19)	N1—C13—N2	113.56 (16)
C5—C4—H4	119.2	N1—C13—C12	126.81 (16)
C3—C4—H4	119.2	N2—C13—C12	119.43 (16)
C4—C5—C6	116.54 (18)	C12—C14—H14A	109.5
C4—C5—H5	121.7	C12—C14—H14B	109.5
C6—C5—H5	121.7	H14A—C14—H14B	109.5
C5—C6—N2	132.18 (17)	C12—C14—H14C	109.5
C5—C6—C1	122.82 (17)	H14A—C14—H14C	109.5
N2—C6—C1	104.98 (15)	H14B—C14—H14C	109.5
N2—C7—C8	113.43 (14)	C16—C15—C20	118.52 (18)
N2—C7—H7A	108.9	C16—C15—C11	121.05 (15)
C8—C7—H7A	108.9	C20—C15—C11	120.42 (17)
N2—C7—H7B	108.9	C15—C16—C17	120.30 (18)
C8—C7—H7B	108.9	C15—C16—H16	119.8
H7A—C7—H7B	107.7	C17—C16—H16	119.8
C9—C8—C7	106.26 (13)	C18—C17—C16	120.76 (19)
C9—C8—C11	109.21 (14)	C18—C17—H17	119.6
C7—C8—C11	114.35 (15)	C16—C17—H17	119.6
C9—C8—H8	109.0	C17—C18—C19	119.40 (19)
C7—C8—H8	109.0	C17—C18—H18	120.3
C11—C8—H8	109.0	C19—C18—H18	120.3
O2—C9—O3	124.16 (16)	C20—C19—C18	120.24 (19)
O2—C9—C8	123.49 (16)	C20—C19—H19	119.9
O3—C9—C8	112.31 (14)	C18—C19—H19	119.9
O3—C10—H10A	109.5	C19—C20—C15	120.77 (19)
O3—C10—H10B	109.5	C19—C20—H20	119.6
H10A—C10—H10B	109.5	C15—C20—H20	119.6
O3—C10—H10C	109.5	C13—N1—C1	104.27 (15)
H10A—C10—H10C	109.5	C13—N2—C6	106.69 (14)
H10B—C10—H10C	109.5	C13—N2—C7	126.10 (15)
O1—C11—C15	107.31 (14)	C6—N2—C7	127.21 (15)
O1—C11—C8	112.11 (13)	C11—O1—C12	116.85 (14)
C15—C11—C8	110.49 (14)	C9—O3—C10	116.93 (15)
			
N1—C1—C2—C3	−176.13 (17)	C20—C15—C16—C17	−0.4 (3)
C6—C1—C2—C3	1.1 (3)	C11—C15—C16—C17	−179.17 (17)
C1—C2—C3—C4	−0.3 (3)	C15—C16—C17—C18	−0.5 (3)
C2—C3—C4—C5	−0.6 (3)	C16—C17—C18—C19	0.9 (3)
C3—C4—C5—C6	0.6 (3)	C17—C18—C19—C20	−0.4 (3)
C4—C5—C6—N2	177.99 (19)	C18—C19—C20—C15	−0.5 (3)
C4—C5—C6—C1	0.2 (3)	C16—C15—C20—C19	0.9 (3)
N1—C1—C6—C5	176.63 (16)	C11—C15—C20—C19	179.70 (17)
C2—C1—C6—C5	−1.1 (3)	N2—C13—N1—C1	0.64 (19)
N1—C1—C6—N2	−1.64 (19)	C12—C13—N1—C1	−174.24 (17)
C2—C1—C6—N2	−179.41 (16)	C2—C1—N1—C13	178.12 (19)
N2—C7—C8—C9	168.67 (16)	C6—C1—N1—C13	0.66 (19)
N2—C7—C8—C11	−70.8 (2)	N1—C13—N2—C6	−1.7 (2)
C7—C8—C9—O2	57.1 (2)	C12—C13—N2—C6	173.61 (16)
C11—C8—C9—O2	−66.7 (2)	N1—C13—N2—C7	178.08 (16)
C7—C8—C9—O3	−120.72 (16)	C12—C13—N2—C7	−6.6 (3)
C11—C8—C9—O3	115.47 (16)	C5—C6—N2—C13	−176.11 (19)
C9—C8—C11—O1	178.71 (14)	C1—C6—N2—C13	1.94 (19)
C7—C8—C11—O1	59.82 (18)	C5—C6—N2—C7	4.1 (3)
C9—C8—C11—C15	−61.64 (17)	C1—C6—N2—C7	−177.83 (16)
C7—C8—C11—C15	179.47 (14)	C8—C7—N2—C13	64.6 (2)
O1—C12—C13—N1	114.80 (19)	C8—C7—N2—C6	−115.64 (19)
C14—C12—C13—N1	−4.2 (3)	C15—C11—O1—C12	164.34 (14)
O1—C12—C13—N2	−59.8 (2)	C8—C11—O1—C12	−74.17 (18)
C14—C12—C13—N2	−178.84 (17)	C13—C12—O1—C11	90.09 (18)
O1—C11—C15—C16	−139.65 (16)	C14—C12—O1—C11	−148.12 (16)
C8—C11—C15—C16	97.85 (19)	O2—C9—O3—C10	0.7 (3)
O1—C11—C15—C20	41.6 (2)	C8—C9—O3—C10	178.51 (17)
C8—C11—C15—C20	−80.90 (19)		
